# Beyond microbial exposure and colonization: multisensory shaping of the gut microbiome

**DOI:** 10.1128/msystems.01107-25

**Published:** 2025-09-24

**Authors:** Jake M. Robinson, Martin F. Breed

**Affiliations:** 1 College of Science and Engineering, Flinders University, Bedford Park, South Australia, Australia; 2 The Aerobiome Innovation and Research Hub, Flinders University, Bedford Park, South Australia, Australia; Qingdao Institute of Bioenergy and Bioprocess Technology, Qingdao, Shandong, China

**Keywords:** gut microbiome, gut–brain axis, epigenetics, microbiota, multisensory

## Abstract

Microorganisms play a fundamental role in human health, contributing to digestion, immune regulation, and metabolic processes. While direct colonization by environmental microbes through ingestion, inhalation, and dermal contact has been documented, evidence suggests that multisensory interactions with nature—via visual, auditory, tactile, gustatory, and olfactory stimuli—also influence the gut microbiome through psychophysiological and immune-mediated pathways. Exposure to natural environments can regulate stress and immune responses, activate the parasympathetic nervous system, and modulate the hypothalamic–pituitary–adrenal and gut–brain axes, which in turn may alter gut microbiome composition and function. Furthermore, sensory interactions with nature may induce epigenetic changes that impact immune function and microbiome dynamics over time. Here, we review evidence for nature-based indirect shaping of the human microbiome (including multisensory and exposure-immunoregulation pathways) and suggest that after the early-life critical window of microbiome development (0–3 years), these indirect effects likely have a greater influence on gut microbiome dynamics than direct colonization by environmental microbiota (e.g., ingested directly from the air). However, this concept remains to be comprehensively tested. Therefore, understanding the relative contributions of direct microbial colonization versus indirect effects—such as multisensory stimulation and immune modulation—demands more integrated, transdisciplinary research. Integrating these insights into public health strategies, urban design, and nature-based interventions could promote microbiome eubiosis, ultimately improving human (and non-human animal) well-being in an era of increasing environmental and health challenges.

## THE MICROBIOME AND US: A MULTIDIMENSIONAL CONNECTION

Human health is deeply intertwined with the natural environment, with microbial exposure playing a critical role in maintaining physiological and psychological homeostasis (1). Exposure to environmental microbes (particularly via dietary and antenatal pathways) during the critical microbiome developmental window (i.e., approximately 0–3 years of age) is known to shape gut microbiota composition, which is associated with health and behavior outcomes (2–4). For instance, maternal fecal microbiota colonize the guts of newborns, at least transiently, and further development of the child’s gut microbiome is influenced by the mode of nutrient intake, for example, during breastfeeding (5, 6). However, human–environment interactions that impact our microbiome extend far beyond direct microbial transfer and colonization.

Multisensory experiences of nature—including visual engagement with fractal patterns, listening to diverse bird songs, tactile interaction with bark and soil, gustatory exploration of natural foods, and the olfactory detection of natural volatiles—can profoundly influence human well-being (7–10). These interactions can reduce stress, enhance immunoregulation, and activate the parasympathetic nervous system, promoting relaxation and modulating the body’s endocrine, immune, and central nervous systems (11) along key biological axes, including the hypothalamic–pituitary–adrenal (HPA) axis (12) and, likely, the gut–brain axis. Moreover, exposure to environmental stressors (e.g., chemical, noise, and light pollution) and neuro-esthetically displeasing stimuli (e.g., degraded, unsafe environments, and some buildings) can increase allostatic load, that is, the cumulative physiological and psychological burden placed on the body due to chronic exposure to stressors and the resultant activation of stress response systems (13–15).

**Fig 1:**
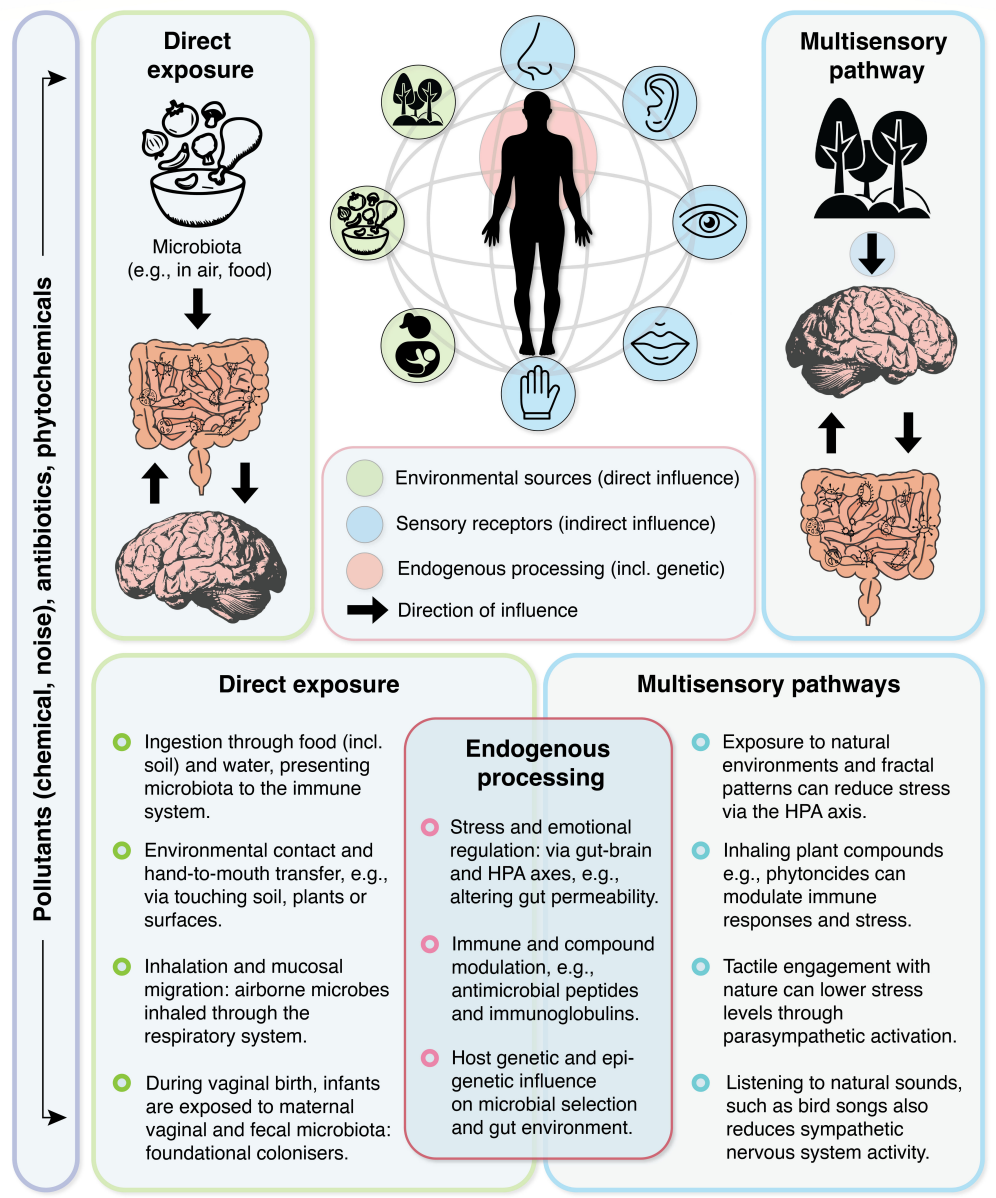
An integrative framework. The multidimensional shaping of the gut microbiome: sensory reception and endogenous processing play a key role (in addition to direct exposure by microbiota and biochemical input from pollutants and phytochemicals).

In theory, these multisensory interactions—in addition to endogenous processing (e.g., emotional regulation via the limbic system or gene expression changes through epigenetic mechanisms)—can shape the human gut microbiome, including the gut environment and the composition and functional roles of the resident microbiota (16). Therefore, a threefold pathway—(i) direct microbial colonization, (ii) immune and biochemical responses to environmental exposures, and (iii) multisensory experiences—likely links the environment to gut health and dynamics. This necessitates the development of an integrative framework to understand the relative importance of these three pathways and potential synergistic effects, aiming to inform research and interventions designed to leverage nature-based but also simply everyday experiences for human health (Fig. 1).

## DIRECT COLONIZATION BY ENVIRONMENTAL MICROBES

We consume between 6 × 10^6^ (6 million) and 1.3 × 10^9^ (1.3 billion) bacteria daily (17). Direct microbial colonization involves the transfer of environmental microbes to the human microbiome through ingestion, inhalation, or dermal contact. The gut microbiota is most malleable during early life, making this a critical window for microbial establishment (18). While soil, plants, and unprocessed foods may be key sources of beneficial microbes, evidence supporting direct and long-term microbial colonization throughout the life course is limited.

Brown et al. (19) investigated how gardening might influence the human gut microbiome, comparing families who gardened with those who did not. They observed increases in microbial diversity and a higher relative abundance of some fiber-fermenting taxa in gardeners, alongside an average overlap between adult fecal and soil microbial communities of 3.2%—tested via fast expectation-maximization microbial source tracking (FEAST). The findings support at least transient microbial transfer from the soil to the gut. However, the evidence is not strong for long-term colonization. Limitations include small sample size, use of 16S rRNA sequencing (which lacks species- and strain-level resolution), and reliance on self-reported diet data (19).

Mouse model studies have reported the transfer of environmental microbiota to the gut. For instance, one study offers evidence that exposure to soil environments can alter the composition of the gut microbiota in mice and influence immune function (20). Mice housed with soil exhibited higher Bacteroidetes/Firmicutes ratios and upregulation of immunoregulatory genes like IL-10 and Foxp3. Some soil-associated bacterial taxa were detected in the intestines of these mice, indicating microbial transfer. However, while these findings support microbial transfer and modulation of host immunity, they do not definitively demonstrate long-term colonization of environmental microbes; most detected taxa in these animals were present at low abundance and not found consistently across individuals. Another study showed that airborne microbes from biodiverse soils can transfer to mouse guts and, at least temporarily, alter microbial composition. However, it does not confirm long-term colonization, as the microbes’ persistence after exposure was not assessed (and 16S rRNA sequencing is unable to determine viability or activity) (21).

Direct colonization likely depends on factors such as dietary substrates, host genetics, and competition with—and nutrient blocking by—existing microbiota (22). Microbial species adapted to environmental niches, such as soil-derived *Bacillus* strains, may, at least, transiently colonize the gut, providing metabolic and immunological benefits. However, more research is needed to determine long-term colonization potential by environmental microbiota, particularly after the early-life critical window of microbiome development. This also applies to the skin microbiome, as most studies have shown only transient colonization or have not tested for long-term establishment. For instance, the number of bacterial taxa shared between skin and garden soil can increase immediately after gardening. However, the imprint of these microbes largely disappears within 12 h (23).

## MULTISENSORY EXPERIENCES AND INDIRECT PATHWAYS

Indirect influences on the gut microbiome are under-explored and operate through immunomodulatory pathways, the psychophysiological effects of multisensory interactions with nature, and endogenous processing, such as the body’s internal responses to thoughts and memories.

From an immunological perspective, rural environments, typically with high “natural” microbial biodiversity, correlate with reduced population rates of allergies, autoimmune diseases, and metabolic disorders. For example, children raised in traditional farming environments show enhanced immune function (e.g., protection against asthma and allergic sensitization) compared to their urban and mechanized/chemical-based farming counterparts (24, 25), which is associated with local environmental and fecal microbiota (26). Moreover, microbial transfer via biodiversity interventions is associated with diversified skin microbiomes and enhanced immune regulation in children (27, 28). Specifically, biodiversity interventions may diversify both the environmental and skin Gammaproteobacterial communities and increase plasma TGF-β1 levels, the proportion of regulatory T cells, and the plasma IL-10:IL-17A ratio.

The immune system is also increasingly exposed to synthetic compounds and particles that were not present during the majority of humans’ recent evolutionary journey (e.g., tetrachlorodibenzo-o-dioxins, perfluorinated compounds, polychlorinated biphenyls, microplastics, and metal nanoparticles). While research on their long-term effects remains limited, attention has only recently turned to how these exposures may influence early-life development. Researchers suggest that microplastics may carry pathogens and disrupt the human immune system (29). A recent pilot study found that microplastics—detected in human blood—were associated with changes in gut microbial species and their functions (30). Using metagenomic analysis of human stool and mouse models, the researchers showed that microplastic exposure correlated with increases in genes related to microbial virulence, quorum sensing, transporter systems, and microplastic degradation. Notably, beneficial species like *Faecalibacterium prausnitzii* were reduced in individuals with higher microplastic levels. These findings suggest that microplastics may impact gut microbiome functionality, with implications for human health.

Noise and light pollution may also affect both the immune system and gut microbiota (31). For instance, chronic noise exposure (100 dB, 400 Hz–6.3 kHz, 4 h daily for 30 days) was found to shift the relative abundance of Proteobacteria and Actinobacteria in the guts of rats, alongside disruptions in glucose and insulin regulation compared to control animals (32). Prolonged exposure to airport noise can significantly increase blood pressure in mice and lead to marked alterations in the structure and composition of their gut microbiota (33). Low-level road traffic noise and individual noise sensitivity may impact human health by modulating immune function through pathways that are independent of cortisol elevation (34). One study also found that higher average daily noise levels are associated with increased COVID-19 cases, hospitalizations, and ICU admissions, via immune and stress-related pathways (35). It is, therefore, feasible that the environmental buffering effects of biodiverse green and blue spaces (36) may have a considerable modulating role on the human microbiome by reducing exposure to these pollutants.

**Fig 2:**
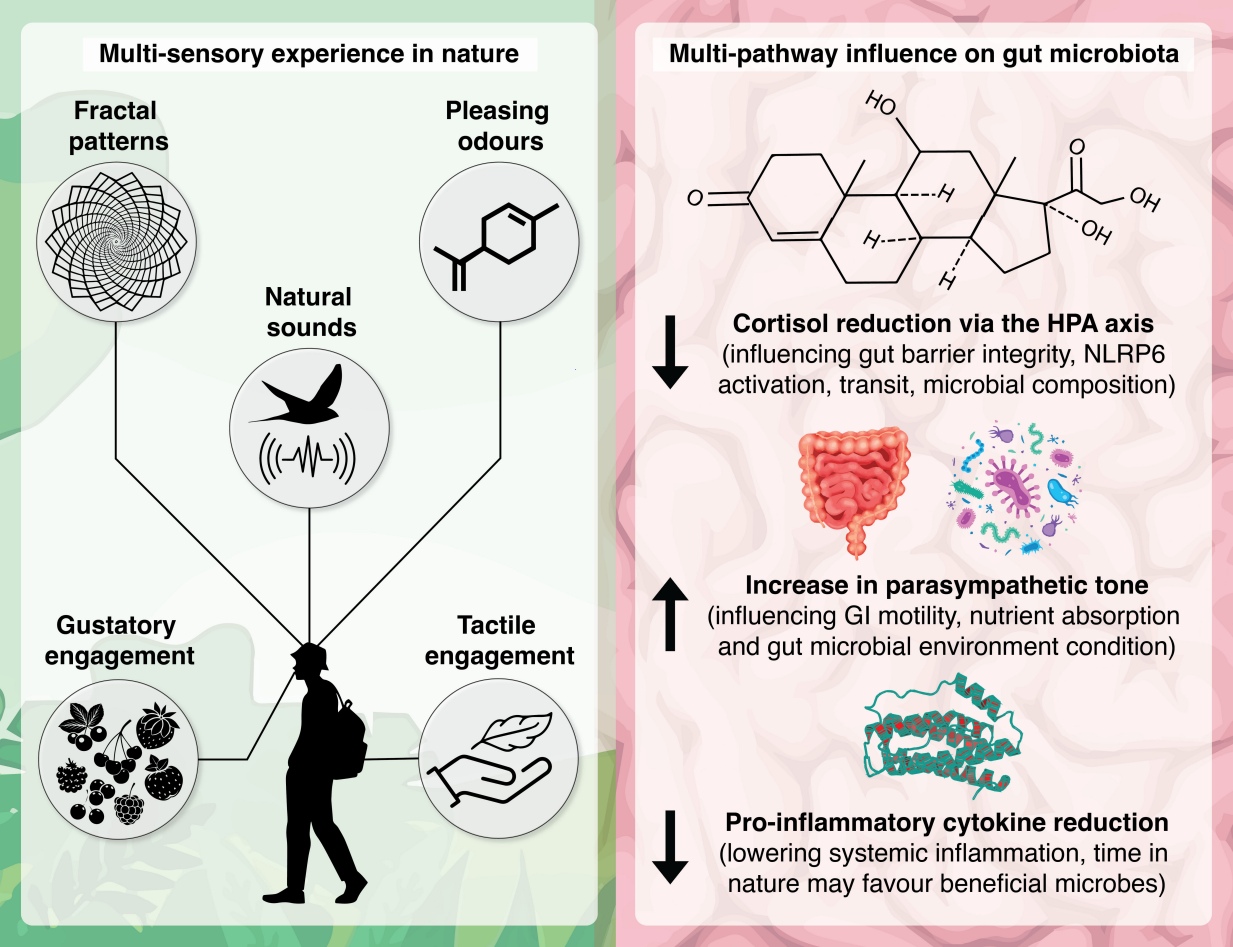
Beyond direct microbial colonization: multisensory experiences in nature and their potential to indirectly influence the gut microbiome via multiple biological systems and axes. For example, stress can impair gut immunity in part by downregulating NLRP6 activity, a key regulator of the inflammasome and microbial balance (42); elevated cortisol increases gut permeability (43), and nature walks (and listening to nature sounds) reduce cortisol (44) and increase parasympathetic tone (45, 46). Exposure to natural biodiverse materials can reduce pro-inflammatory cytokines (27, 28). All of these processes can influence the gut environment, composition, and dynamics.

From a multisensory perspective, visual, auditory, tactile, gustatory, and olfactory inputs influence stress physiology, immune modulation, and behavioral patterns, likely indirectly shaping the gut environment, microbiota, and dynamics (Fig. 2). Visual exposure to, and mindful engagement with, biodiverse green spaces can reduce stress hormone levels (e.g., cortisol) and enhance parasympathetic nervous system activity (7). Such effects are likely to result in changes in the HPA and gut–brain axes—the latter being the bidirectional communication system linking the central nervous system with the gastrointestinal tract. This cascade influences gut microbiome dynamics, including gut barrier integrity, transit time, nutrient absorption, and microbial composition (16). There is considerable research linking exposure to and engagement with natural environments to reduced allostatic load (37, 38) and on the crosstalk between the HPA axis and gut microbiome (39–41). While more research is clearly needed to explore the stress-regulating effects of nature exposure on the microbiome, the theoretical support is strong.

Natural environments also release volatile organic compounds, such as phytoncides and geosmin, which activate olfactory receptors. Some of these compounds (e.g., α-pinene, β-pinene, limonene, 1,8-cineole [eucalyptol], linalool, borneol, and camphene) are associated with reduced sympathetic nervous system activity, enhanced emotional well-being and positive affect, immune regulation (e.g., NK cell activity) (47), reduced diastolic and systolic blood pressure (48), and potentially improved sleep—for example, by stimulating the GABAA-benzodiazepine receptors (49), all of which are associated with the microbiota–gut–brain axis (1). Tactile engagement with natural surfaces, such as plant materials, listening to pleasing natural sounds (e.g., melodic bird calls and a gentle wind blowing through leaves), and mindfully eating elicit stress-reducing effects by inducing parasympathetic nervous activity and calming prefrontal cortex activity (50). Multisensory experiences in nature can also reduce pro-inflammatory cytokines. This can lead to reduced systemic inflammation, potentially favoring the conditions for salutogenic (health-promoting) gut microbes to proliferate.

Nature exposure may also influence the gut microbiome indirectly through epigenetic pathways, though these mechanisms remain underexplored. For instance, cortisol can affect DNA methylation of stress-related genes (51), which may influence immune function and gut microbiota composition. Exposure to natural environments has also been linked with enhanced cognitive function (52), which may entail epigenetic mechanisms, potentially impacting gut–brain communication. Emerging evidence suggests that epigenetic changes induced by environmental exposures can also be transmitted across generations. The potential indirect effects of transgenerational inheritance on the gut microbiome warrant further study.

Moreover, while host epigenetics is increasingly recognized in microbiome studies, the epigenetic modifications within microbial communities themselves, such as DNA methylation, may dynamically influence their functional output, host interactions, and broader health effects (53). This microbial epigenetic flexibility may be crucial for maintaining a resilient and functional gut ecosystem. Moreover, the crosstalk between microbial and host epigenetics suggests a bidirectional “epigenome–microbiome axis,” where environmental factors can influence both microbial and host gene expression, potentially affecting health outcomes (54). For instance, *Lactobacillus* and *Bifidobacterium* species produce folate, which supports DNA methylation and mRNA N6-methyladenosine (m6A) modification in the gut, processes essential for normal intestinal development (55).

Together, these processes highlight the limitations of host-centric models of health and illustrate the so-called “holobiont blindspot” (56)—the tendency to overlook how the host and its microbial partners, through both genomic and epigenomic crosstalk, co-regulate physiological outcomes in response to environmental stimuli.

## A FRAMEWORK TO INTEGRATE DIRECT AND INDIRECT PATHWAYS

We propose a conceptual framework that integrates direct microbial colonization, exposure-immunoregulation pathways, multisensory interactions, endogenous processing, and epigenetic regulation to explain how environmental exposures and experiences influence the gut microbiome (Fig. 1). This framework sits on three foundations: (i) synergistic effects, that is, direct exposure to microbes and indirect sensory experiences often occur simultaneously (for example, gardening involves tactile microbial transfer [57] alongside stress-reducing sensory stimuli [58] [there are also stress-reducing microbes in soil associated with gardening {59}]); (ii) context dependency, that is, factors such as life stage, baseline health, and environmental context may modulate the relative importance of direct and indirect pathways (for instance, direct microbial transfer is crucial during early life, while adults may benefit [relatively] more from multisensory and other indirect mechanisms); and (iii) feedback loops (for example, positive interactions with nature can enhance behaviors that promote microbial exposure, such as outdoor activities and dietary choices).

## RESEARCH AND POLICY IMPLICATIONS

To advance our understanding in this field, we recommend addressing the following research questions as a priority.

- Relative contributions: what are the relative effects of direct colonization versus multisensory experiences (and other indirect pathways) on the gut (and other body sites) microbiome?
- Mechanistic pathways: how do sensory experiences modulate human microbiomes via the gut–brain axis and epigenetic regulation?
- Intervention design: can interventions that combine direct microbial exposure and multisensory nature engagement produce synergistic benefits?
- Epigenetic insights: what are the specific epigenetic markers influenced by environmental microbial exposure and multisensory experiences, and how do they relate to health outcomes?

Considering the multisensory experiences and other indirect pathways in shaping the gut microbiome has profound implications for public health, urban planning, and environmental policy. Policies must move beyond pathogen-focused frameworks to embrace the salutogenic potential of nature-based interventions (60). Encouraging innovative greenspace designs in urban planning can provide communities with accessible opportunities for multisensory engagement with nature, promoting stress reduction, immune modulation, and potentially improved gut health. Additionally, integrating evidence-based nature prescriptions (61) into healthcare systems could complement traditional treatments, targeting chronic stress-related illnesses and promoting holistic well-being strategies. Policymakers should also prioritize the protection and restoration of biodiverse ecosystems, which likely serve as reservoirs for beneficial microbes and sensory stimuli. We should also consider the multisensory experience of mindfully eating food. For instance, does where, how, when, and what we eat shape our moods, thereby triggering biological interactions that indirectly influence our microbiomes? From this perspective, policymakers could incorporate mindfulness into nutrition education. Finally, cross-sector collaborations between environmental scientists, urban planners, and public health experts are essential to creating environments that support the multiple pathways of microbial exposure, colonization, and multisensory engagement, ensuring sustainable health benefits for diverse populations.
